# Gartner’s Duct Cyst of the Vagina: A Case Report

**DOI:** 10.31729/jnma.5009

**Published:** 2020-07-31

**Authors:** Baburam Dixit Thapa, Mohan Chandra Regmi

**Affiliations:** 1 Department of Obstetrics and Gynecology, B. P. Koirala Institute of Health Sciences, Dharan, Nepal; 2 Department of Obstetrics and Gynecology, B. P. Koirala Institute of Health Sciences, Dharan, Nepal

**Keywords:** *muellerian ducts*, *pelvic organ prolapses*, *wolffian ducts*

## Abstract

Paramesonephric duct or Mullerian ducts forms female genital organs whereas mesonephric duct forms male genital organs. The remnant of the mesonephric duct or Wolffian duct in females sometimes forms a mesonephric cyst or Gartner's duct cyst. They are usually asymptomatic and <2 cm but sometimes can be bigger. It is diagnosed with pelvic examination. It is treated with surgical excision of the cyst. This is a unique case in urogynecology as it confuses with pelvic organ prolapse and the mode of treatment is completely different. We report a case of 32-years old lady who presented in urogynecology outpatient department with complain of pelvic organ prolapse. After examination she was diagnosed as vaginal cyst and excision was done and confirmed as Gartners cyst in histopathological examination.

## INTRODUCTION

Wolffian duct forms the male genital tract. In females, they regress. Occasionally they remain and the caudal portion forms the vaginal inclusion cyst known as Gartner duct cyst.^1^ They are mostly asymptomatic and are found incidentally during the pelvic examination. Other common vaginal cysts include epidermal inclusion cyst, Bartholin duct cyst, and Muellerian cyst. Gartner duct cyst is frequently associated with other renal congenital abnormalities.^2^

They are usually less than <2 cm but can present with bigger mass as well.^3^ We report a Gartner duct cyst which was approximately 6 cm and presented with mass coming out per vaginum.

She was non-diabetic, non-hypertensive, non-smoker, and no such history in her family members. She showed in the local hospital and was diagnosed as pelvic organ prolapse (cystocele) and referred to our centre. She has no other complaint of discharge per vaginum, urinary retention, urinary incontinence, bowel problems, and bleeding per vaginum.

**Figure 1.**
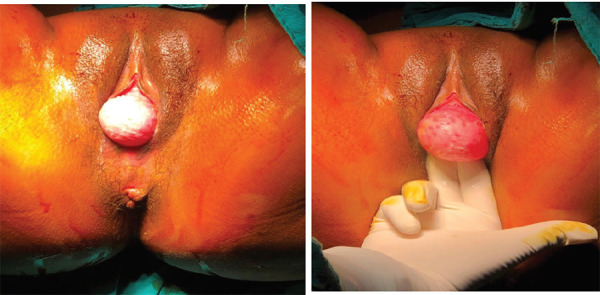
Gartner duct cysts.

On per speculum examination, there was a cystic, nontender, mobile mass of 6 × 6 cm mass in the suburethral region, protruding from the left posterolateral vaginal wall (Figure 1).

## CASE REPORT

A 32-year para 2, living 2, last childbirth 7 months back, presented with complaints of mass coming out per vaginum for 4 months which was progressive. She first noticed it 4 months back when it was small in size. She delivered both babies vaginally and there was no history of difficult, prolonged, or instrumental delivery.

Cervix was normal. The uterus was normal size and adnexa was free and non-tender bilaterally. We ruled out other diagnoses of Bartholin's duct cyst, cystocele with the examination. The endometriotic cyst was ruled out with no other significant symptoms, the free pouch of Douglas and normal uterus, and ovaries. We did a transvaginal ultrasound to rule out urethral diverticulum. It showed a single cyst of 6 × 6 cm, with no connection to the urethra. We also did cystourethroscopy to rule out urethral diverticulum with zero degrees cystoscope and 30-degree cystoscope to rule out bladder diverticulum.

The provisional diagnosis of the Gartner duct cyst was made. Her routine blood investigations and urinalysis was normal. Transabdominal ultrasound was done to rule out any abdominal and genitourinary abnormalities. Magnetic resonance imaging (MRI) could have been done but it was not done as it was expensive and don't add much in our diagnosis and further management.

The patient was posted for surgical excision and cystectomy was done in a lithotomy position under spinal anesthesia. The cyst was sent for histopathology. It came as non-mucin secreting low columnar and cuboidal epithelium, consistent with the Gartner duct cyst. The patient was followed up after 2 weeks and 3 months. She did not have any complaints then.

## DISCUSSION

Gartner's duct cyst may present with mass coming out per vaginum. General vaginal examination and transvaginal ultrasound are sufficient for diagnosis. Surgical excision is the mainstay of treatment if they are symptomatic and large. MRI is more diagnostic but not needed in most of the cases.^2–4^

In female Mullerian ducts form the female genital system whereas Wolffian duct regress and form a vestigial system.^4^ This forms the Gartner duct cyst and is typically located in the anterolateral vaginal wall following the course of the duct.^5^ They are usually asymptomatic but may cause dyspareunia, mass coming out per vaginum and urinary symptoms.^4^ MRI is more diagnostic but are not recommended as it is the expensive and general pelvic examination and transvaginal ultrasound are sufficient for management.^3^ Malignant transformation of cyst is very rare.^6^ Though surgical excision is the mainstay of treatment, it can be managed expectantly if it is asymptomatic and small.^7^ In our case the cyst was large so we did surgical excision. Diagnosis is confirmed with histopathology which shows cyst lined with a non-mucinous cuboidal or low columnar epithelium. Smooth muscles may be seen around the Gartner duct cyst in pathological examination.^8^

## Consent:

**JNMA Case Report Consent Form** was signedby the patient and the original article is attached withthe patient's chart.

## Conflict of Interest

**None.**
